# Brain Healthcare Quotient as a Tool for Standardized Approach in Brain Healthcare Interventions

**DOI:** 10.3390/life14050560

**Published:** 2024-04-26

**Authors:** Keitaro Yoshida, Kiyotaka Nemoto, Ami Hamano, Masahito Kawamori, Tetsuaki Arai, Yoshinori Yamakawa

**Affiliations:** 1 Department of Psychiatry, Division of Clinical Medicine, Institute of Medicine, University of Tsukuba, Tsukuba 305-8575, Japan; keicima0154@gmail.com (K.Y.); hamano.ami.mp@alumni.tsukuba.ac.jp (A.H.); 4632tetsu@md.tsukuba.ac.jp (T.A.); 2 Graduate School of Media and Governance, Keio University, Fujisawa 252-0882, Japan; kawamorim@gmail.com; 3 ImPACT Program of Council for Science, Technology and Innovation (Cabinet Office, Government of Japan), Chiyoda, Tokyo 102-0076, Japan; yamakawa@bi-lab.org; 4 BRAIN IMPACT General Incorporated Association, Kyoto 606-8501, Japan; 5 Office of Society-Academia Collaboration for Innovation, Kyoto University, Kyoto 606-8501, Japan; 6 Institute of Innovative Research, Tokyo Institute of Technology, Meguro, Tokyo 152-8550, Japan; 7 Office for Academic and Industrial Innovation, Kobe University, Kobe 657-8501, Japan

**Keywords:** brain healthcare, aging, MRI, Brain Healthcare Quotient, intervention

## Abstract

In addressing the challenge of assessing healthy brain aging across diverse interventions, this study introduces the use of MRI-derived Brain Healthcare Quotients (BHQ) for comprehensive evaluation. We analyzed BHQ changes in 319 participants aged 24–69, who were allocated into dietary (collagen peptide, euglena, matcha, isohumulone, xanthophyll) and physical activity (hand massage with lavender oil, handwriting, office stretching, pink lens, clinical art) groups, alongside a control group, over a month. These interventions were specifically chosen to test the efficacy of varying health strategies on brain health, measured through BHQ indices: GM-BHQ for gray matter volume, and FA-BHQ for white matter integrity. Notably, significant improvements in FA-BHQ were observed in the collagen peptide group, with marginal increases in the hand massage and office stretching groups. These findings highlight BHQ’s potential as a sensitive tool for detecting brain health changes, offering evidence that low-intensity, easily implemented interventions can have beneficial effects on brain health. Moreover, BHQ allows for the systematic evaluation of such interventions using standard statistical approaches, suggesting its value in future brain healthcare research.

## 1. Introduction

As the older population increases, healthcare and social welfare burdens will rise [1], as evidenced by projections from the United States that indicate Social Security and Medicare deficits of at least US$52 trillion over the next 30 years due to chronic diseases of aging in the “Baby Boom” generation [2]. In the face of the global challenges of population aging and an unprecedented crisis from the increase in age-related neurodegenerative diseases, experts have sought a paradigm shift in brain health, shifting from “treatment of dysfunction” to “maintenance of health and prevention of dysfunction” [3]. Thus, health authorities have strengthened efforts to better understand healthy brain aging and its determinants, identify interventions that promote healthy brain aging, and translate the research to create and disseminate sustainable public health programs [4].

Although some progress has been made in modifiable risk factors and corresponding preventive approaches, current evidence remains modest and inconclusive [5] due to heterogeneity across studies of interventions (e.g., types of exposures and/or outcomes, length of follow-ups, biases and/or confounders that were accounted for, and overall quality of studies) and difficulty in quantifying outcomes. This lack of standardization renders accurate assessment of preventive interventions and comparisons of impact across studies difficult, as well as preventing replication of previous findings [6]. For example, frequently used exposure variables, such as body mass index, waist circumference, quantity/years of tobacco consumption, amount of alcohol intake, blood pressure level, total cholesterol, and fasting glucose, are often categorized differently across studies. In terms of outcome measures, studies rarely use common variables. The World Alzheimer Report 2014 [7] showed that the following diverse outcome measures have been used in previous studies: cognitive outcomes, mild cognitive impairment, dementia, composite measures of cognition, cognitive activity, incident dementia, activities of daily living, executive function, memory, reasoning, processing speed, attention, memory, Alzheimer’s Disease Assessment Scale-cognitive subscale, and hippocampal volume. The heterogeneity of these elements prevents meta-studies of available evidence, and best-practice advisement is stalled in the face of conflicting and imprecise data. Thus, the World Alzheimer Report 2014 called for a consistent and standardized approach for future studies [7].

In 2017, we proposed a standardized magnetic resonance imaging (MRI)-based quotient for monitoring brain health based on analyses of gray matter volume (the Brain Healthcare Quotient based on gray matter volume [GM-BHQ]) and fractional anisotropy of white matter (FA-BHQ). GM-BHQ represents the gray matter volume as a standardized score, taking into account that the degree of atrophy in gray matter varies by region. Likewise, FA-BHQ represents the integrity of white matter considering the regional differences of FA. We found that both GM- and FA-BHQs were highly sensitive to age-related declines in the brain, and both were significantly associated with physical factors (e.g., obesity and blood pressure), lifestyle factors (e.g., daily schedules), and social factors (e.g., subjective socioeconomic status, subjective well-being, and post-materialism) [8]. Other studies indicate that GM-BHQ is positively associated with curiosity [9] or motivation [10], yet it shows negative correlations with factors like fatigue [11]. Similarly, FA-BHQ is positively linked to subjective happiness [12], but it is inversely associated with anxiety [13]. Considering these findings, the BHQ has the potential to serve as a marker for various interventions due to its associations with diverse factors. Therefore, in the present study, we extend this standardized BHQ outcome approach to evaluate the effectiveness of interventions for brain healthcare.

## 2. Materials and Methods

### 2.1. Participants

A total of 330 healthy participants (30 participants per intervention and 30 participants as control), aged 20–69 and with no significant medical histories or contraindications of MRI, were recruited from local cities in Hyogo, Kyoto, and Tokyo areas in Japan between October 2015 and December 2017. All participants gave written, informed consent prior to participation. This study conformed to the principles of the Declaration of Helsinki and was approved by the Kyoto University Institutional Review Board (approve number 27-P-13).

### 2.2. Diet and Activity Interventions

The present study focuses on evaluating the role of the following two categories of interventions in maintaining brain health: diet/nutrition and regular physical activity. We publicly chose brain healthcare interventions to evaluate the effectiveness of BHQs as an assessment tool. After a number of companies and organizations were recruited, a selection committee composed of experts in brain healthcare evaluated the feasibility and potential of each candidate intervention. Finally, the following 5 dietary and 5 physical activity interventions were selected based on expert analyses: collagen peptides, euglena, matcha (powdered green tea), isohumulone, and xanthophylls for dietary interventions, and hand massage with lavender oil, handwriting, office stretching, pink lens, and clinical art for physical activity interventions.

In terms of dietary interventions, the Mediterranean diet has been associated with improved cognitive function, and studies have also explored the connection between physical activity and dementia [14]. The participants were recruited and assigned to one of the 10 intervention groups or a control group (See Table 1 for descriptive statistics). The ten total intervention groups consisted of 5 dietary intervention groups (collagen peptide, euglena, matcha, isohumulone, or xanthophyll) and 5 physical activity intervention groups (hand massage with lavender oil, handwriting, office stretching, pink lens, or clinical art).

For the dietary intervention groups, all participants underwent an MRI scan, took a specified amount of the test supplements daily for approximately a month, and then underwent a second MRI scan. For the physical activity intervention groups, all participants also underwent a first MRI scan, performed the assigned physical activity for specified durations of time or at specified frequencies (daily or weekly) for approximately a month, and then underwent a second MRI scan. Participants who could not complete the intervention were excluded. As a result, 319 participants (168 males and 151 females) were analyzed. A summary of the experimental conditions for each of the 10 interventions is provided in Table 1. Randomization was not performed when assigning these participants as the focus was to evaluate the usefulness of BHQ as an assessment tool for a standardized approach rather than to rigorously evaluate the effectiveness of each intervention.

### 2.3. Imaging Procedures

#### 2.3.1. MRI Data Acquisition

All magnetic resonance imaging (MRI) data were collected using a 3-T Siemens scanner (Verio, Siemens Medical Solutions, Erlangen, Germany or MAGNETOM Prisma, Siemens, Munich, Germany) with a 32-channel head array coil at Kyoto University, the University of Tokyo, and RIKEN.

High-resolution structural images were acquired using a three-dimensional (3D), T1-weighted, magnetization-prepared, rapid-acquisition gradient echo (MP-RAGE) pulse sequence using the following parameters for Kyoto University and the University of Tokyo (Verio): repetition time (TR), 1900 ms; echo time (TE), 2.52 ms; inversion time (TI), 900 ms; flip angle, 9°; matrix size, 256 × 256; field of view (FOV), 256 mm; and slice thickness, 1 mm. For RIKEN (Prisma), parameters were the following: repetition time (TR), 2000 ms; echo time (TE), 2.13 ms; inversion time (TI), 952 ms; flip angle, 9°; matrix size, 256 × 256; field of view (FOV), 230 mm; and slice thickness, 0.9 mm.

DTI data were collected via spin-echo echo-planar imaging (SE-EPI) with GRAPPA (GeneRalized Autocalibrating Partially Parallel Acquisitions). The image slices were parallel to the orbitomeatal (OM) line. The parameters were as follows for Kyoto University and the University of Tokyo (Verio): TR, 141,00 ms; TE, 81 ms; flip angle, 90°; matrix size, 114 × 114; FOV, 224 mm; and slice thickness, 2 mm. A baseline image (b = 0 s/ mm^2^) and 30 different diffusion orientations were acquired with a b value of 1000 s/mm^2^. For RIKEN (Prisma), parameters were the following: TR, 3700 ms; TE, 82 ms; flip angle, 90°; matrix size, 118 × 118; FOV, 200 mm; and slice thickness, 1.7 mm. Data was acquired in two shells as follows: six baseline images (b = 0 s/ mm^2^) and 54 different diffusion orientations were acquired with b values of 1000, 2000, and 3000 s/mm^2^.

#### 2.3.2. Image Preprocessing

T1-weighted images were preprocessed and analyzed using Statistical Parametric Mapping 12 (SPM12; Wellcome Trust Centre for Neuroimaging, London, UK) running on MATLAB R2020b (Mathworks Inc., Sherborn, MA, USA). Gray matter (GM) images were extracted, spatially normalized using the diffeomorphic anatomical registration through exponentiated lie algebra (DARTEL) algorithm [15], and then smoothed with an 8-mm full width at half-maximum (FWHM) Gaussian kernel. Intracranial volume (ICV) was also calculated from the results of segmentation. Proportional GM images were generated by dividing smoothed GM images by ICV to control for differences across participants. Using these proportional GM images, mean and standard deviation (SD) images were generated from all participants. We calculated the GM-BHQ images using the following formula for each voxel: 100 + 15 × (individual proportional GM − mean)/SD. Regional GM quotients were then extracted using an automated anatomical labeling (AAL) atlas [16] and averaged across regions to produce participant-specific GM-BHQs.

Diffusion data were preprocessed using the FMRIB Software Library (FSL) 6.0.4 [17]. First, all diffusion images were aligned with the initial b0 image, and motion correction and eddy current distortion correction was performed using eddy_correct. Following these corrections, FA images were calculated using dtifit. FA images were then spatially normalized into the standard Montreal Neurological Institute (MNI) space using FLIRT and FNIRT. Normalized data were smoothed with an 8-mm FWHM. Mean and SD images were generated from all the FA images. Individual FA quotient images were calculated using the following formula for each voxel: 100 + 15 × (individual FA − mean)/SD. Regional FA quotients were extracted using Johns Hopkins University (JHU) DTI-based white-matter atlases [18] and averaged across regions to produce participant-specific FA-BHQs. Nemoto et al. [13] showed that both GM-BHQ and FA-BHQ reflect age-related declines in GM volume and WM integrity, verifying BHQ as a highly sensitive tool for evaluating intervention outcomes. After carrying out GM-BHQ and FA-BHQ for pre-treatment (Pre) and post-treatment (Post) tests, GM-BHQ and FA-BHQ gain scores were computed by subtracting GM-BHQ pre-treatment scores from GM-BHQ post-treatment scores and by subtracting FA-BHQ pre-treatment scores from FA-BHQ post-treatment scores.

Note that as each participant used the same MRI scanner twice and we measured only differences between the two timepoints, MRI machine differences were judged to have no impact on the results.

### 2.4. Statistical Analyses

We employed R 3.6.0 [19] for the statistical analysis. The current study employed analysis of covariance (ANCOVA) of gain scores to compare changes in GM- and FA-BHQ from pre-intervention to post-intervention between the experimental conditions. As mentioned, 10 experimental conditions (interventions) were divided into two groups (i.e., dietary component, physical activity), and compared separately within each group. Age and the duration of time between Pre and Post were included in all analyses as control variables. Post hoc pairwise comparisons were performed by adjusting *p*-values using the Benjamini–Hochberg procedure [20] to control the False Discovery Rate (FDR) for multiple comparisons.

#### Removal of Outliers

The original data appeared to be non-normal due to a few extreme outliers. Thus, we performed outlier analyses using the median absolute deviation (MAD). The MAD has been suggested as an alternative to and the most robust measure of dispersion compared to more popular methods, such as mean plus or minus a coefficient (2, 2.5, or 3) times standard deviations and the classical interquartile range [21].

We calculated the MADs (1.539 and 1.162, respectively for GM- and FA-BHQ gain scores) and the decision criteria for both GM-BHQ (−4.754, 4.478) and FA-BHQ (−3.470, 3.502) gain scores following the procedure proposed by Leys et al. [21]. Thus, all GM-BHQ gain score values smaller than −4.754 and greater than 4.478, plus all FA-BHQ gain score values smaller than −3.470 and greater than 3.502, were removed as outliers. The normality checks after removing these outliers show that the distributions for both GM-BHQ gain and FA-BHQ gain scores were normalized.

## 3. Results

### 3.1. One-Way ANCOVA on GM-BHQ Gain Scores for Dietary Components

**Figure 1:**
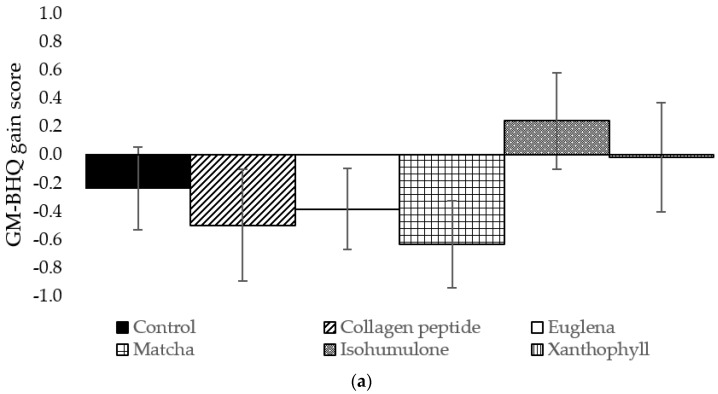

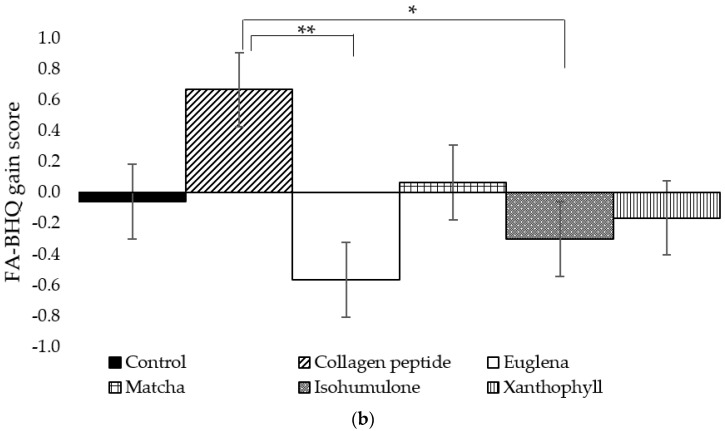
(**a**) GM-BHQ gain scores for dietary component groups. (**b**) FA-BHQ gain scores for dietary component groups. Values are expressed as estimated marginal means ± standard errors. * Benjamini–Hochberg adjusted *p* < 0.05 compared to isohumulone group; ** Benjamini–Hochberg adjusted *p* < 0.01 compared to euglena group.

A one-way, between-participants ANCOVA was conducted to compare the effect of dietary components on GM-BHQ gain scores, controlling for the effect of age and the duration of time between Pre and Post. There were no significant differences in the effects of any dietary components on GM-BHQ gain scores, F (5, 159) = 1.114, *p* = 0.355 (Figure 1a).

### 3.2. One-Way ANCOVA on FA-BHQ Gain Scores for Dietary Components

A one-way, between-participants ANCOVA was conducted to compare the effect of dietary components on FA-BHQ gain scores, controlling for the effect of age and the duration of time between Pre and Post. There was a statistically significant difference between the effect of dietary components on FA-BHQ gain scores: F (5, 156) = 3.195, *p* = 0.009 after controlling for the effect of age and the duration of time between Pre and Post. Both age and the duration of time between Pre and Post were not significant covariates: F (1, 156) = 0.653, *p* = 0.420, and F (1, 156) = 0.010, *p* = 0.921, respectively.

Post hoc tests showed that the collagen peptide group had significantly higher FA-BHQ gain scores than the euglena (Benjamini–Hochberg adjusted *p* = 0.008) and isohumulone groups (Benjamini–Hochberg adjusted *p* = 0.028) (Figure 1b).

### 3.3. One-Way ANCOVA on GM-BHQ Gain Scores for Physical Activities

**Figure 2:**
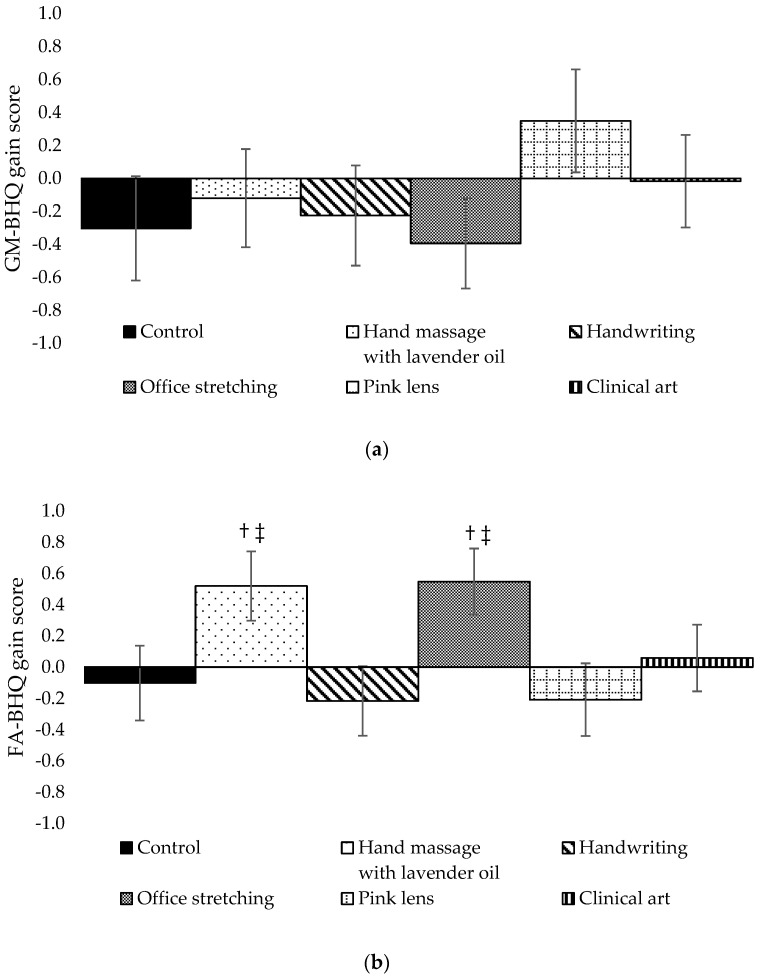
(**a**) GM-BHQ gain scores for physical activity groups. (**b**) FA-BHQ gain scores for physical activity groups. Values are expressed as estimated marginal means ± standard errors. † Benjamini–Hochberg adjusted *p* < 0.10 compared to handwriting group. ‡ Benjamini–Hochberg adjusted *p* < 0.10 compared to pink lens group.

A one-way, between-participants ANCOVA was conducted to compare the effect of physical activities on GM-BHQ gain scores, controlling for the effect of age and the duration of time between Pre and Post. There were no significant differences in the effects of any physical activities on GM-BHQ gain scores, F (5, 166) = 0.799, *p* = 0.552 (Figure 2a).

### 3.4. One-Way ANCOVA on FA-BHQ Gain Scores for Physical Activities 

A one-way, between-participants ANCOVA was conducted to compare the effect of physical activities on FA-BHQ gain scores, controlling for the effect of age and the duration of time between Pre and Post. There was a statistically significant difference in the effect of physical activities on FA-BHQ gain scores: F (5, 163) = 2.752, *p* = 0.020, controlling for the effect of age and the duration of time between Pre and Post. Both age and the duration of time between Pre and Post were not significant covariates: F (1, 163) = 3.188, *p* = 0.076, and F (1, 163) = 2.511, *p* = 0.115, respectively. 

Post hoc tests showed that no significant differences between groups after adjusting *p*-values for multiple comparison though the differences between the following groups remained marginally significant (Figure 2b): hand massage with lavender oil and handwriting groups (Benjamini–Hochberg adjusted *p* = 0.081), hand massage with lavender oil and pink lens groups (Benjamini–Hochberg adjusted *p* = 0.081), office stretching and handwriting groups (Benjamini–Hochberg adjusted *p* = 0.081), and office stretching and pink lens groups (Benjamini–Hochberg adjusted *p* = 0.081) (Figure 2b).

## 4. Discussion

In this study, we showed that different interventions could be evaluated if we use BHQ. Though the differences between physical activities were only marginally significant, FA-BHQ values for the hand massage with lavender oil group and office stretching group did increase while FA-BHQ values for three of the other four groups, including the control group, decreased. Therefore, it may be possible that the differences between the former two groups and the latter three groups would increase to the extent that differences in imaging results would become significant if participants continued for longer periods of time or performed these activities more frequently or at greater intensity.

On the other hand, there were no significant differences between both dietary and physical activity groups for GM-BHQ values. It is difficult to determine the possible reasons why these dietary components and physical activities appear to work somewhat better on FA-BHQ than GM-BHQ. However, one possible explanation may be the fact that GM-BHQ tends to deteriorate faster as we age than FA-BHQ, as previously demonstrated by the stronger correlation between GM-BHQ and age compared to the correlation between FA-BHQ and age [13]. If GM-BHQ values typically decrease faster than FA-BHQ as we age, dietary components and physical activities with a greater effect in slowing or warding off age-related decline may compensate for the decline. Another possible explanation may be that dietary components and physical activities may have a different effect on different parts of the brain. Further studies that elucidate these fine details will add resolution to the overall brain health picture regarding BHQ measurements.

We believe that BHQs have several advantages over common measures used in research in this arena. First, the use of measures of brain structure and/or integrity is essential in brain healthcare or dementia prevention research, as previous longitudinal studies have shown that brain alterations may be present long before cognitive decline and/or neurodegenerative disorders are clinically expressed [22,23,24,25,26,27,28,29,30]. Second, as progression from normal state to mild cognitive impairment (MCI) to full-blown dementia is a steady and slow process that can extend over decades [31], measuring the intervention effect may be difficult. By using BHQs, even early and silent stages of age-related brain alterations and/or brain diseases may be identified, and the effect of interventions more rapidly evaluated. Third, conventional MRI techniques typically rely on visual analysis and require considerable expertise to accurately identify abnormalities in the brain. Even for trained experts, subtle changes in the brain can be overlooked until the changes are discernible to the human eye [32]. On the other hand, by using standardized BHQs, even subtle changes across time could be more easily identified and systematic comparisons between patients, facilities, and/or organizations would be possible using common statistical techniques [32]. Accumulation of such large-scale data would also prove invaluable for training artificial intelligence to support diagnoses and evaluations. Fourth, BHQ scores are simple to understand, even for lay people, and could become a popularly known criterion for public health agencies to use in promoting preventative care programs.

Despite strenuous efforts to develop countermeasures for neurodegenerative diseases, the impact of simple, short-term, inexpensive, and easy-to-implement low-intensity interventions cannot currently be fully evaluated. The effects of these low-intensity interventions may be relatively small compared to moderately intense, expensive, or complex medical/surgical interventions; however, frequency and intensity may compensate for lower impact. Thus, BHQ is a crucial tool for evaluating interventions, regardless of participant age and intervention complexity, and the standardized data will allow for direct comparison. This will potentially save on healthcare costs due to encouraging the early adoption of simple interventions (as is currently done with heart disease and stroke), as well as simplify a complex concept to facilitate public education. Moreover, utilizing a standardized numerical index, such as BHQ, may help strengthen evidence-based practices by allowing accumulation of big data that can be analyzed (i.e., through meta-analysis) to detail the relationships between frequencies and degrees of intensity of interventions and resulting effects. This could apply to the development of both treatment and preventive interventions and lead to best practice development in clinical and public health sectors. 

It is important to note that the current study was conducted using a non-randomized, controlled design. Though each ANCOVA was controlled for the effect of age and the duration of time between Pre and Post, other unknown confounding factors (e.g., current health status, regular dietary intake, regular physical activity, socio-economic status) may still have been present, limiting the conclusions regarding the true relationship between these interventions and brain alterations. Therefore, cause–effect relationships cannot be established based solely on the results of this study, and the results must be interpreted with caution. Longitudinal, randomized, controlled trials are needed to confirm the findings of this study and to find the true relationships between various interventions for brain healthcare and subsequent brain changes. However, the present study contributes to the literature on brain healthcare by showing that the recently developed, standardized index, BHQ, is useful as it catalogs subtle changes in the brain and distinguishes slight differences between interventions in terms of those changes.

## Figures and Tables

**Table 1:** Descriptive Statistics by Group.

Groups	N (Male/Female)	Age (M ± SD)	Interval (Days) between Pre–Post (M)	Quantity/Frequency
Control	30 (15/15)	53.9 ± 9.3	28.0	-
Collagen peptide	29 (25/4)	55.6 ± 4.2	28.2	5 g/day
Euglena	30 (14/16)	47.8 ± 6.8	30.0	1 g/day
Matcha	27 (11/16)	48.1 ± 5.8	28.4	2 g/day
Isohumulone	25 (14/11)	57.3 ± 6.2	27.8	13.2 mg/day
Xanthophyll	30 (15/15)	48.8 ± 5.2	33.4	9 mg/day
Hand massage with lavender oil	30 (0/30)	35.6 ± 4.7	31.6	10 min/3 times/week
Handwriting	30 (23/7)	41.2 ± 11.0	28.0	5 min/day
Office stretching	30 (22/8)	45.6 ± 9.9	32.6	5 min/day
Pink lens	29 (0/29)	43.8 ± 6.0	35.7	3 h/day
Clinical art	39 (29/0)	48.3 ± 5.5	29.7	1 h/week

## Data Availability

The data presented in this study are available upon request from the corresponding author.
